# Exploring the Dynamic of Bacterial Communities in Manila Clam (*Ruditapes philippinarum*) During Refrigerated Storage

**DOI:** 10.3389/fmicb.2022.882629

**Published:** 2022-05-18

**Authors:** Yi Yang, Jingxuan Qiu, Xin Wang

**Affiliations:** School of Health Science and Engineering, University of Shanghai for Science and Technology, Shanghai, China

**Keywords:** Manila clam, microbiota, bacterial diversity, high-throughput sequencing, refrigerated storage

## Abstract

Microorganism contamination is one of the most important factors affecting the spoilage and food safety of Manila clams. This study aimed to gain insights into bacterial composition and the dynamic change of bacterial communities on retailed Manila clam during refrigerated storage within the edible period. High-throughput sequencing was conducted to monitor the bacterial population with the prolongation of storage time of Day 0, Day 1, and Day 3. Result demonstrated that phyla of Proteobacteria, Actinobacteriota, Acidobacteriota, and Chloroflexi composed the majority of bacterial communities during the whole observation process. Furthermore, the increase of Proteobacteria showed a positive correlation with the storage time, whereas Acidobacteriota and Chloroflexi continued to decline in storage. For genus annotation, none of genus obtained dominant population in storage. From Day 0 to Day 1, the genera of *Streptomyces*, *Bradyrhizobium*, and *Mycobacterium* significantly increased; meanwhile, 12 genera significantly decreased. Compared with samples at Day 0, a total of 15 genera significantly decreased with the reduced proportion ranging from 0.50 to 4.40% at Day 3. At the end of the storage, the genus *Crossiella* became the most redundant population. Both the richness and diversity decreased at the start of storage at Day 1, and then slightly increased at Day 3 was observed. Based on the result in this study, strategy targeting the increased bacteria could be tested to improve the consumption quality and safety of refrigerated clam.

## Introduction

The Manila clam, *Ruditapes philippinarum*, is one of the important and most exploited marine bivalves around the world; it is nutritious containing different kinds of proteins, vitamins, and essential elements (Zhao and Zhang, 2016). Due to the great ability on adapting environment, Manila clam reproduced rapidly and distributed widely. Nowadays, Manila clam has become the world’s second most important commercially cultured bivalve mollusk (Tan et al., 2020). China is the largest producer of Manila clam, about 3 million tons of clams were produced each year, which is almost 90% of the world’s production (Nie et al., 2016). Nevertheless, just like the most shellfish, Manila clam always suffer from the contamination of diverse microorganisms, due to the abundant water and nutrient contents (Chen et al., 2019). The proliferation of microorganisms may lead to the short shelf-life and unacceptable qualities of clam (Boziaris and Parlapani, 2017). During the transportation and storage, low-temperature storage is the most common approach to preserve the fresh clam. Thus, in order to improve the consumption quality and safety of refrigerated clams, it is essential to investigate the dynamics of contaminating bacterial communities in clam during refrigerated storage.

Among the approaches in microorganism identification, two types of methods including culture-dependent microbial cultivation and culture-independent high-throughput sequencing method have been applied. For culture-based methods, since only 0.1–3% of the bacteria could be cultivated (Amann et al., 1995; Cocolin et al., 2013), the experiment data obtained from cultivating bacteria are insufficient to clarify the microbiota of food (Caporaso et al., 2012). With the development of high-throughput sequencing technology, molecular methods, which based on sequence characterization, could provide a comprehensive description of the whole microbiota. The variable region on 16S rRNA gene was used as the indicator for taxonomy identification. Polymerase chain reaction (PCR) cloning, combined with 16S rRNA gene sequencing could provide high-throughput bacterial identification with high efficacy and low cost (Klindworth et al., 2013), and the technology was applied widely on evaluating the bacterial composition of seafood, during the storage period (La Valley et al., 2009; Trabal et al., 2012; Chen et al., 2016, 2019), with different processing technology (Cao et al., 2018) and with different package (Pan et al., 2018). For example, the microbiome of the fresh clam from different geographic origin was identified using 16S rRNA gene sequencing, and the abundance of Proteobacteria with Acidobacteria could be used as a marker in distinguishing different geographic origin of fresh clam (Liu et al., 2020). Through 16S rRNA gene sequencing, genera *Psychrobacter* and *Psychromonas* were identified as the most important spoilage organisms of oysters during refrigerated storage (Chen et al., 2019). The 16S rRNA gene amplicon sequencing of bacteria on mussel revealed that Proteobacteria, Cyanobacteria, and Firmicutes were the three major phyla in modified atmosphere packed mussel (Odeyemi et al., 2019). Apart from detecting the microbial diversity in seafood, high-throughput sequencing has a wide application in several food products, revealing the bacterial or fungal composition. For example, the bacterial community in zha-chili, fermented by different rice varieties, was detected using high-throughput sequencing (Cai et al., 2021a). It was found that rice variety would lead to different microbiota in fermented zha-chili sample, in which lactic acid bacteria obtained the most dominant abundance of 77.09% (Cai et al., 2021a). The microorganism structure, including bacterial richness and fungal diversity, in low-temperature Daqu for Baijiu-making was also been explored (Cai et al., 2021b). The core microbiota influencing the flavor of Daqu were dominated by *Thermoactinomyces*, *Lactobacillus*, *Saccharopolyspora*, along with *Bacillus*, *Streptomyces*, *Saccharomycopsis*, and *Thermoascus* (Cai et al., 2021b). Pit mud is the important carrier for various microorganism in the production of Chinese Baijiu (Cai et al., 2022). Through Illumina MiSeq sequencing, the fungal community was analyzed, and diverse high-abundance fungi in pit mud at different depths was revealed, which could be useful in improving the quality of pit mud (Cai et al., 2022). All of the above studies illustrated that 16S rRNA gene sequencing could provide a rapid illustration of whole bacterial communities. The identified bacterial profile could provide a theoretical basis on improving food safety and maintain food quality. Nevertheless, few studies have been reported to identify the bacterial change in refrigerated clam, especially during edible shelf-life storage.

In this study, the V3–V4 regions of bacterial 16S rRNA gene on retailed Manila clam were sequenced to monitor the dynamic change of microorganism composition during refrigerated storage. The bacterial abundance at different taxonomy annotation level was investigated, and the change of bacterial diversity was identified. The result in this study could provide a hint on risk assessment for clams during process and storage.

## Materials and Methods

### Raw Material Acquisition and Sample Preparation

Philippine clams originated from Dandong City, Liaoning Province, refrigerated and transported from terminal to the Shanghai distributor, and delivered to the school laboratory *via* the online purchase platform of Dingdong in 0.5–1 h, during which the clams were transported in low-temperature brine to ensure quality. The clams were cleaned in the laboratory with sterilized water for surface debris such as mud, sand, and dirt, after which they were drained and stored in the refrigerator at 4 ± 1°C sealed with food wrap. Based on previous microbial early experiments and sensory analysis, the clams with similar size at Day 0, Day 1, and Day 3 were divided into three groups (D 0, D 1, and D 3). Each group is divided into three parallel samples (D 0_1, D 0_2, and D 0_3), (D 1_1, D 1_2, and D 1_3), and (D 3_1, D 3_2, and D 3_3), respectively, and the shell meat was removed aseptically in a sterile ultra-clean table and cut with a sterilized knife to ensure the samples were evenly placed into 10-ml centrifuge tubes and frozen at −80°C.

### DNA Extraction, Amplification, and Sequencing

The total genomic DNA of microbial colonies from clams was extracted by E.Z.N.A. Soil DNA Kit (Omega Bio-tek, Norcross, GA, United States). The 1% agarose gel electrophoresis was used to detect DNA samples. The purity of DNA was assessed by NanoDrop 2000 UV-Vis Spectrophotometer (Thermo Scientific, Wilmington, DE, United States).

The V3–V4 regions of bacterial 16S rRNA genes in clams were amplified by PCR with a primer pair of 338F (5′-ACTCCTACGGGAGGCAGCAG-3′) and 806R (5′-GGACTACHVGGGTWTCTAAT-3′). The amplification procedure included an initial denaturation step (95°C for 3 min) followed by 27 cycle reactions composing denaturation (95°C for 30 s), annealing (55°C for 30 s), extension (72°C for 30 s), and continuous extension at 72°C for 10 min. PCR products were extracted from 2% agarose gel electrophoresis and purified using AxyPrep DNA Gel Extraction Kit (Axygen Biosciences, Union City, CA, United States).

A DNA library was constructed using NEXTflex Rapid DNA-Seq Kit (Bioo Scientific, United States) according to the following steps: (1) adding adapter sequences; (2) removing the fragments connected by different adapters; (3) enrichment of library templates using PCR amplification; and (4) collecting PCR products.

Purified amplicons were pooled in equimolar, and paired-end sequencing was conducted on an Illumina MiSeq PE300 Platform (Illumina, San Diego, CA, United States) according to the standard protocols by Majorbio Bio-Pharm Technology Co., Ltd. (Shanghai, China).

### Processing of Sequence Data

At first, the raw sequencing reads were filtered by fastp version 0.20.0 (Chen et al., 2018). Using a sliding window of 50 base pair (bp), the 300-bp reads for which the average quality score were less than 20 were truncated. After truncating, sequences longer than 50 bp were remained. Sequencing reads which included ambiguous characters were also abandoned.

Second, filtered sequences with overlapping fragments longer than 10 bp were merged by FLASH version 1.2.7 (Magoc and Salzberg, 2011). In overlapping fragment, the maximum proportion of mismatch ratio was set as 0.2. Reads that could not be assembled were also discarded.

The barcodes and primers added in library construction were used to distinguish different samples, with the following criteria: (1) exact barcode matching and (2) less than 2 mismatches in primer matching.

Operational taxonomic units (OTUs) were clustered by UPARSE version 7.1 (Edgar, 2013) to represent sequences with similarity greater than or equal to 97% (Stackebrandt and Goebel, 1994; Edgar, 2013). The taxonomy assignment of representative sequences for each OTU was annotated by RDP Classifier version 2.2 (Wang et al., 2007) at different levels, using confidence threshold of 0.7.

### Statistical Analysis

To test sequence quantity, Shannon rarefaction curves (Caporaso et al., 2010) were applied to test whether the amount of sequenced reads were enough to reflect the bacterial community in each sample. Different indices, including Sobs, Shannon, Simpson, Ace, and Chao1 (Amato et al., 2013), were calculated to estimate the diversity of microbial communities in each sample. Index of Good’s coverage was calculated to evaluate sequencing depth. In order to detect OTU distribution, a Venn diagram was used to reflect the overlapped and unique species in samples at different storage time (Li et al., 2017). Based on the bacterial composition in each sample, hieratical clustering analysis were conducted to test the bacterial similarities. Principal component analysis (PCA) was used to select the discriminated composition variables. Between two different sample groups, bacterial genus with significantly different (*p* < 0.05) proportion were selected by Student’s *t*-test (Al Ashhab et al., 2014). To detect the change of bacterial community abundance among multiple groups, linear discriminant analysis effect size (LEfSe) analysis was used to screen the differentially distributed features. At first, the non-parametric factorial Kruskal–Wallis (KW) test rank-sum test was used to screen genera with significant abundance difference. And then linear discriminant analysis (LDA) score of each bacterial genus was calculated as the quantitative estimator of effects on sample grouping; the cutoff of LDA was set as 2 (Segata et al., 2011).

## Results

### Microbial Communities Detected by 16S rRNA Gene Sequencing

**FIGURE 1:**
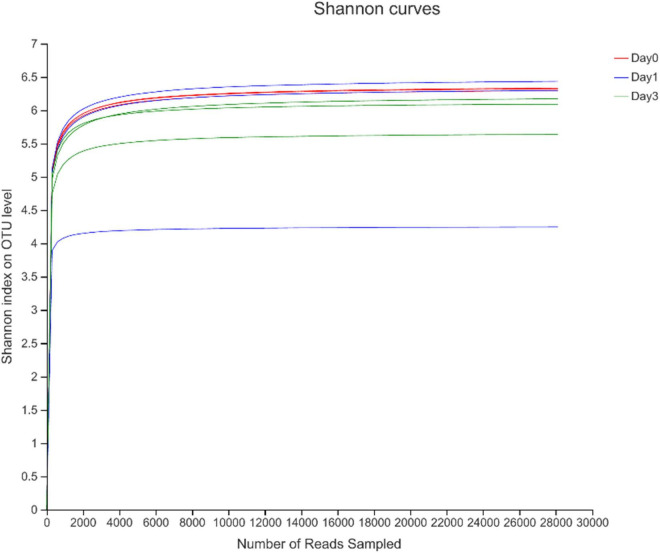
Rarefaction analysis of high-throughput sequencing reads in samples at different storage time.

To describe the character of bacterial community of clams at different refrigerated storage time, V3–V4 regions on 16S rRNA gene were sequenced by MiSeq high-throughput sequencing. After quality filtering, a total number of 306,675 reads were obtained in all samples. The detailed information of sequencing is shown in Table 1. Among them, each sample contained 34,075 ± 7,648.08 (mean ± SD) sequences. The sample of D 3_3 contained the maximum sequence number of 49,646. To avoid the bias caused by different sequence numbers in each sample, effective tags were randomly selected to reach the same amount of reads. The sequence number is set as 28,130 in D 0_3 sample, which is the minimum sequence number among all samples. For all samples, the average length of sequenced reads reached 415 bp. Then 4,883 OTUs were clustered in all samples, each cluster represented a group of similar sequences, and these OTUs were annotated to 40 phyla, 131 classes, 313 orders, 489 families, 893 genera, and 1,815 species. During refrigerated storage, the average number of OTUs decreased from 2,059.67 at Day 0 samples to 1,681 and 1,695.67 at Day 1 and Day 3 samples, respectively. The change of OTUs illustrated the variance in bacterial composition in different samples. The results of rarefaction analysis showed that with the increase of randomly sampled sequences, Shannon index curves for each sample all resulted in a platform stage (Figure 1). The result demonstrated that the sequencing quantity in each sample could well reflect the structure and diversity of bacterial communities. The Good’s coverage values calculated in each sample were all higher than 99.68%, which also indicated that the sequence obtained in the current study is sufficient to represent the actual bacteria species.

**TABLE 1:** Effective sequence numbers, OTUs, and Good’s coverage of clam meat during refrigerated storage at 0, 1, and 3 days.

Sample group	Sample name	Reads	OTU	Good’s coverage (%)
Day 0	D 0_1	30,341	2,060	98.18
	D 0_2	30,433	2,021	98.28
	D 0_3	28,130	2,098	98.08
Day 1	D 1_1	44,576	519	99.46
	D 1_2	31,422	2,299	97.89
	D 1_3	30,347	2,225	98.02
Day 3	D 3_1	33,282	2,246	97.75
	D 3_2	28,498	1,541	98.89
	D 3_3	49,646	1,300	99.04

### Analysis of Bacterial Community Diversity and Richness

To illustrate the diversity of bacterial communities, different estimators were introduced. The index of Sobs, Ace, and Chao1 at OTU level (Supplementary Table S1) and genus level (Table 2) for each sample was calculated to illustrate the community richness. In Table 2, it is found that all samples achieved the highest values for all these three measures at Day 0, indicating that samples at Day 0 obtained the most abundant bacteria. The richness estimator decreased at Day 1 and slightly increased at Day 3. It could also be seen that bacteria at Day 1 contained great variance within the group, which was caused by the data bias at D 1_1 sample. Shannon and Simpson indices were used to indicate the alpha diversity of bacterial communities in clam samples at different refrigeration time; the greater of Shannon index and the smaller Simpson index all referred to the great community diversity. From these two indices, it is demonstrated that during storage, the diversity started to decrease at Day 1 and then increased at Day 3, indicating the communities obtained a more even distribution with less dominant bacteria.

**TABLE 2:** Alpha diversity analysis at genus level.

Diversity estimator	Day 0	Day 1	Day 3
Sobs	513.00 ± 9.64	386.67 ± 136.62	470.33 ± 78.65
Ace	568.11 ± 16.49	422.03 ± 137.44	505.86 ± 74.81
Chao1	573.94 ± 28.86	430.57 ± 137.20	506.26 ± 70.00
Shannon	4.72 ± 0.04	4.52 ± 0.58	4.84 ± 0.17
Simpson	0.018 ± 0.00	0.023 ± 0.01	0.016 ± 0.00

### Bacterial Composition

**FIGURE 2:**
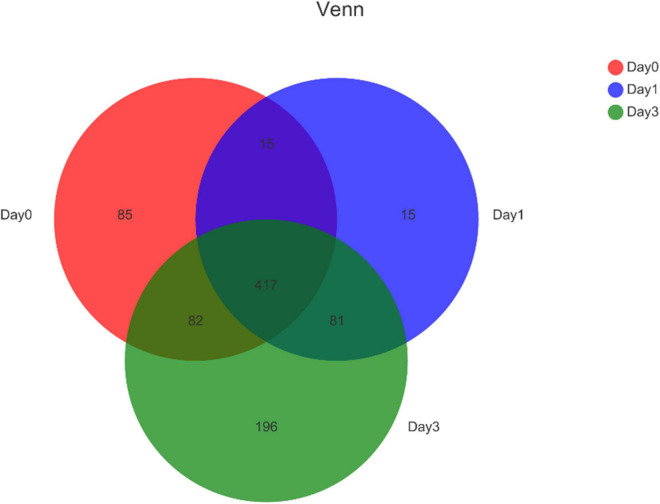
Venn diagram for bacterial genera composition in clam at different storage time. Numbers in different region refers to the number of specific or common genera in different sample group. The sum of numbers in each circle refers to the total genera annotated at each storage time.

Further, the Venn diagram was used to reveal the bacterial distribution of clam at different storage time (Figure 2). In total, 599, 528, and 776 genera were obtained from different samples at Day 0, Day 1, and Day 3, respectively. Among the annotated genera, there were 417 common genera found in all sample groups during the whole storage process. From Day 0 to Day 1, a set of 96 new bacterial genera were emerged, and with storage time increasing, 196 genera were uniquely found at Day 3. The result indicated that even Day 1 shared an 81.82% (432/528) overlap with Day 0, the bacterial community started to change with increased specific species. The communities continued to vary during storage; in the end, samples at Day 3 obtained 35.70% (277/776) specific genera compared to the initial state of Day 0.

**FIGURE 3:**
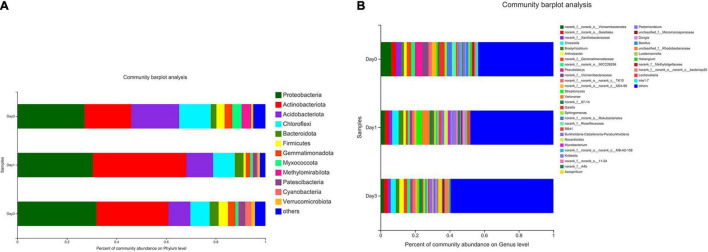
Relative abundance of bacterial composition at phylum and genus levels for samples at different storage time. Each color represents specific phylum **(A)** or genus **(B)**, and the length of each band represents the proportion of particular bacteria.

The composition of bacterial communities during storage process was used to analyze the relative abundance at phylum and genus level (Figure 3). According to Figure 3A, 12 bacterial phyla were found in clam at different storage time. The majority component of phyla were Proteobacteria, Actinobacteriota, Acidobacteriota, and Chloroflexi, composing 77.96, 87.66, and 77.51% of bacterial communities at Day 0, Day 1, and Day 3, respectively. Among them, Proteobacteria accounted for the largest proportion, and the percentage increased during storage, which increased from 27.10% at Day 0 to 31.91% at Day 3, and followed by the phylum of Actinobacteriota, which achieved the abundance of 18.87% at Day 0 and quickly increased to 37.77% at Day 1. On the other hand, Acidobacteriota decreased continuously from 19.27 to 8.81% during storage. Chloroflexi showed a decrease at Day 1 (12.72–8.76%) and maintained a steady abundance afterward. Other phyla were less dominant and included Patescibacteria (0.45–2.05%), Cyanobacteria (0.06–2.48%), and Verrucomicrobiota (0.70–1.43%). Bacteria with a relative abundance less than 1% were labeled as others at phylum and genus levels.

At the genus level (Figure 3B), a total number of 39 genera were observed in clam samples. From the relative abundance of bacterial genera, it should be noted that there was not a dominant genus in each sample group, and even the proportion of the most abundant genus was less than 10%. Then, the detected genera abundance changed greatly during storage. The top five most abundant genera at Day 0 group were *Vicinamibacterales* (6.34%), *Pseudolabrys* (3.97%), *Vicinamibacteraceae* (3.87%), *Xanthobacteraceae* (3.20%), and *Gaiella* (3.18%). At Day 1, the community distribution changed to a different set of genera, including *Variovorax* (4.58%), *Crossiella* (3.76%), *Streptomyces* (3.01%), *Bradyrhizobium* (2.74%), *Vicinamibacterales* (2.57%), and so on. Compared to Day 0, the composition rank changed greatly with different genera at Day 1. At Day 3, bacterial distribution continued to change, for example, the top-ranked bacteria included *Crossiella* (2.94%), *Arthrobacter* (2.60%), *Gaiellales* (2.18%), *Azospirillum* (2.17%), and *Bradyrhizobium* (2.04%). It is demonstrated that the previous top-ranked *Vicinamibacterales* (6.34%) at Day 0 proportioned less than 1% at Day 3, whereas the abundance of *Crossiella* continued to increase during storage. The abundance of the most abundant genus of *Variovorax* (4.58%) at Day 1 decreased to less than 1% at Day 3. From Day 0 to Day 3, the bacterial distribution became more even and more variable, with the primary genus continued to decrease.

**FIGURE 4:**
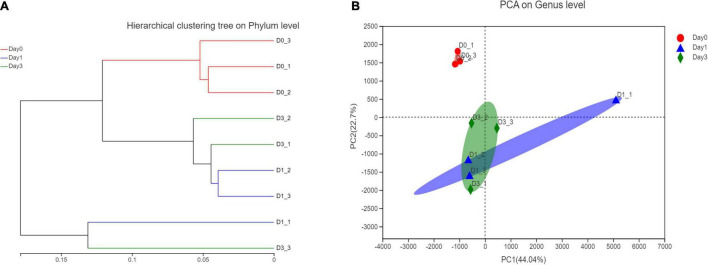
Hierarchical clustering and principle component analysis of bacterial composition in different storage time. **(A)** Hierarchical clustering at phylum level. **(B)** PCA analysis at genus level.

Based on the relative abundance of bacterial genera, a hierarchical clustering tree could be constructed to illustrate the relative similar relationship between different samples. It could be found that samples from Day 0 all clustered together, this result showed the similarity within the group. However, samples from Day 1 to Day 3 showed a close relationship in the tree, indicating the similar bacterial community structure (Figure 4A). To figure out the principal components contributing on sample classification, PCA was performed. In Figure 4B, the cumulative percentage variance for the selected two principle components were 44.04 and 22.7%, composing 66.74% feature contribution. In PCA, samples of Day 0 were separated from samples of Day 1 and samples of Day 3, demonstrating that the bacterial community obtained a significant change, with clam began to spoil since Day 1. For each sample, the deviation of Day 3 from Day 1 illustrated the continuous community variance in the process of storage. Furthermore, the relative location of samples within the same group revealed the bacterial variance in each group, illustrating that the spoiled sample from Day 1 obtained more diverse community composition compared to the closely clustered fresh clam sample at Day 0.

### Microbial Community Changes With Storage Time

**FIGURE 5:**
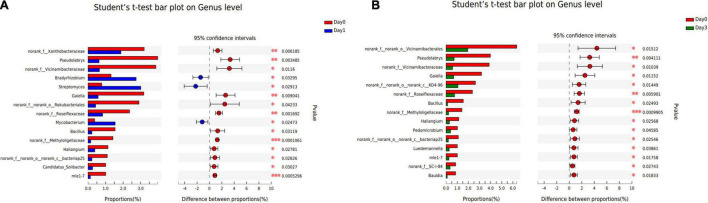
Bacterial genera with significant change of relative abundance between different sample groups. **(A)** Bacterial difference between Day 0 and Day 1. **(B)** Bacterial difference between Day 0 and Day 3.

To illustrate the specific bacterial change between each sample group, two-tailed Student’s *t*-test was used to reveal the significantly changed genera at each storage time (Figure 5). Compared with samples detected at Day 0, three genera were significantly increased at Day 1 with *p*-value less than 0.05, including *Bradyrhizobium*, *Streptomyces*, and *Mycobacterium*. And 12 genera were found to get a significant decrease, in which *Pseudolabrys* obtained the biggest reduction of 3.35%. No genus was found to significantly distribute between Day 1 and Day 3. At Day 3, compared with Day 0, the composition proportion of 15 genera decreased with statistical significance (*p* < 0.05). The reduced proportion ranged from 0.50 to 4.40%. There was no significantly increased genus at Day 3, which is consistent with the result that the bacterial communities reached a more diverse distribution, and each genus obtained a small proportion (see section “Bacterial Composition”).

**FIGURE 6:**
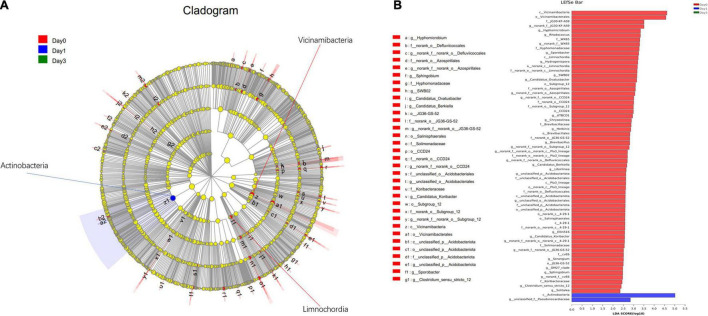
Linear discriminant analysis effect size analysis of differentially distributed genera at Day 0, Day 1, and Day 3 groups. **(A)** From the center to the terminal, each layer refers to phylum, class, order, family, and genus level, respectively. Each node refers to the specific bacteria with node size proportion to the relative abundance. **(B)** Specific bacteria with LDA score more than 2 are displayed.

In addition to the differentially distributed bacterial communities between two groups, genera for which the relative abundance changed significantly in multiple groups were also detected through LEfSe analysis. In Figure 6, the bacterial community with significantly different abundance is observed, which includes *Actinobacteria* class at Day 1, *Vicinamibacteria* class at Day 0, and *Limnochordia* class at Day 0. The results illustrate the significant variance of bacterial composition occurred on the storage from Day 0 to Day 1. LDA score (Figure 6B) showed the abundance of *Actinobacteria* and *Parviterribacter* posed a great effect at Day 1 grouping, with LDA score of 5.01 and 2.85, greater than cutoff of 2. At Day 0 classification, 63 bacteria genera, including *Vicinamibacteria* (LDA score: 4.62), *Vicinamibacterales* (LDA score: 4.57), and so on, could contribute on effective distinguish of Day 0 group.

## Discussion

In this study, bacterial community in *R. philippinarum* was evaluated using high-throughput sequencing of V3–V4 regions on 16S rRNA gene of bacteria. The purpose was to observe the essentials in comprehending the bacterial dynamics in *R. philippinarum* during refrigerated storage. Based on the result, Proteobacteria, Actinobacteriota, Acidobacteriota, and Chloroflexi were the prevalent phyla. With the prolonged storage time, the abundance of Proteobacteria increased steadily and remained at a relative high level, showing a positive correlation with storage time. On the contrary, the phyla of Acidobacteriota and Chloroflexi continued to decline. At the genus level, none of them have a majority species in the population. The abundance of *Vicinamibacterales* and *Pseudolabrys* declined rapidly from Day 1 and continued to decrease in storage. The population of *Crossfield* steadily increased to reach the most redundant genus at Day 3.

Proteobacteria are the largest group of bacteria, and all of the Proteobacteria bacterial communities belong to Gram-negative bacteria. It has been reported that Proteobacteria obtained a high abundance in fresh clam samples harvested from Nova Scotia and Quebec, Canada, and it was a typical spoilage organism for fresh seafood (Liu et al., 2020). Proteobacteria have been detected to dominate the bacterial community in fresh oyster (Madigan et al., 2014; Cao et al., 2018), fresh crisp grass carp (Pan et al., 2018), the gut of grass carp (Shi et al., 2020), frog meat (Yang et al., 2017), mussel meat (Odeyemi et al., 2019), and so on. Furthermore, the processing and storage favored the growth of Proteobacteria in clam (Liu et al., 2020), which is consistent with the observation in this study. Moreover, this phenomenon was also observed in other aquatic meat products, for example, the most dominant phyla of Proteobacteria obtained the high proportion of 77.5% in fresh oysters, with the prolong of refrigerated storage and the process of spoilage, the proportion of Proteobacteria further increased to 88.7% in spoiled oysters (Cao et al., 2018). The relative abundance of Proteobacteria identified from frog meat was 40.29% at Day 0; after storage of 12 days, Proteobacteria become prevalent phyla with abundance of 95.41% (Chen et al., 2021). The similar trend of bacterial abundance in different seafood might illustrate a common spoilage profile, which has been reported that significantly different bacterial composition in different oyster species would end up with similar bacterial profile as oyster spoiled (Madigan et al., 2014). The possible reasons which caused the change of different bacterial community might be related to selective action of different storage conditions and different bacterial activities (Ashie et al., 1996; Naum et al., 2008; Schmitt et al., 2012). Environment factors have been proven to have a crucial role in microbiota, for instance, seasonal variables and exposure to toxicants in environment alerted the composition and metabolic activity of microbiota in fresh Manila clams (Milan et al., 2018), high temperature of seawater might favor the proliferation of opportunistic bacteria in *Mytilus coruscus* (Li et al., 2018), and different sampling year of *Mytilus* also affects the alpha diversity of microbiota (Ramirez et al., 2022). Actinobacteriota was also detected with a great abundantly proportion in clam (Liu et al., 2020), grass carp (Pan et al., 2018; Shi et al., 2020), mussel meat (Odeyemi et al., 2019), and so on. Nevertheless, the abundance rank of Actinobacteriota might be varied in different species (Egerton et al., 2018; Shi et al., 2020) or seafood from different origin (Liu et al., 2020). For Acidobacteriota, the composition proportion of 19.27% at Day 0 sample was observed in this study, and the high abundance of Acidobacteriota in fresh clam was also reported previously (Liu et al., 2020). Even though Acidobacteriota was not the most redundant phylum in bacterial communities, Acidobacteriota often get together with other bacteria to account for a relatively large proportion (Odeyemi et al., 2019; Shi et al., 2020). The occurrences of Chloroflexi in shellfish are rarely reported, and recent studies indicated a decrease of Chloroflexi in seafood at the end of storage time (Odeyemi et al., 2019); in this study, the continuous decline of Chloroflexi from 12.72 to 7.77% during storage was also observed.

Between observed groups at different storage time, several genera with significant change of proportion have been detected, including *Bradyrhizobium*, *Streptomyces*, and *Mycobacterium* between Day 0 and Day 1, *Vicinamibacterales* and *Pseudolabrys* between Day 0 and Day 3, and so on. These significantly distributed genera have also been reported in other aquatic products. For example, *Bradyrhizobium* was occurred in the foot and notum epidermis of nudibranchs (Zhukova et al., 2022). In an investigation of the gut microbial community in olive flounder (*Paralichthys olivaceus*) during different growth stages, *Bradyrhizobium* was composed of the dominant genus at the grower stage of the fish (Niu et al., 2020). It was also been reported that *Bradyrhizobium* obtained the greatest number of sequences in intestinal microbiota from adult pirarucu (*Arapaima gigas*) (Pereira et al., 2017). Among the research of diet-affected changes on gut microbiome of shrimp, *Streptomyces* was one of the most abundant genera on diet-fed group (Prathiviraj et al., 2021). Several genera mentioned in this study could also been served as the probiotics in sustainable aquaculture, for example, *Streptomyces* and *Bacillus* were reported to be good candidates to protect fish and shrimp from pathogens (Tan et al., 2016), or improve feed utilization and disease resistance (Kuebutornye et al., 2019). Besides aquatic products, some genera have also occurred in environment microbiome. For instance, *Vicinamibacterales*, Xanthobacteraceae, and *Pseudolabrys* were all reported to be composed of the soil microbiome in the surface soil (Han et al., 2021); *Panax notoginseng* rhizosphere (Li M. et al., 2020) and *Andrographis paniculata* rhizosphere (Li J. et al., 2020), the abundance of these genera were correlated with the plant yields.

In the clustering and PCA of genus abundance, it is noted that the Day 0 group is separated from Day 1 and Day 3; the clustering result indicated that from Day 0 to Day 1, significant bacterial changes have occurred, followed by the further variation from Day 1 to Day 3. The result is consistent with the statistical result, in which bacterial abundance between Day 0 and other observation time is significantly varied. Meanwhile, no statistical significance can be detected between the genera at Day 1 and Day 3. In the multi-group testing of LEfSe analysis, *Actinobacteria* class had a great impact on grouping of Day 1. Similarly, this class of *Actinobacteria* has also been detected in other seafood, for example, it was reported to occur in the gut microbiota of freshwater fish (Jami et al., 2015) and composed of major bacteria in the gut microbiota of the olive flounder (*P. olivaceus*) (Niu et al., 2020). In the diversity evaluation by high-throughput sequencing data, it could be seen that the indices of Sobs, Ace, and Chao1 illustrate a great variation among the three replications from samples of Day 1. The variation within the group might give a hint on different bacterial community distribution of different tissues or positions from *R. philippinarum*. The position-specific bacterial distribution in aquatic products has been reported, and through 16S rRNA gene sequencing, it is found that the midgut microbiome obtained higher alpha diversity indices than the foregut and hindgut microbiomes in grass carp (Wang et al., 2021) and large yellow croaker (Zhang et al., 2019). The microbiota variance in different organs and sample positions could be taken into consideration in the further study.

## Conclusion

This study provided insights into bacterial composition of Manila clams during refrigerated storage through high-throughput sequencing. The diversity and richness of bacterial communities were evaluated, and the relative abundance of major phyla and genera was calculated based on sequencing result. The detected bacterial change on retailed clam in the process of refrigerated storage could provide theoretical evidence of risk assessment on clam storage. Based on the result, the quality of clam could be evaluated, and different controlling strategies could be tested to prevent the bacterial change.

## Data Availability Statement

The data presented in this study are deposited in NCBI BioProject repository, accession number: PRJNA831322. The repository can be found below: https://www.ncbi.nlm.nih.gov/bioproject/PRJNA831322/.

## Author Contributions

YY conducted the experiments. YY and JQ analyzed the data and wrote the manuscript. XW designed the experiments, modified the manuscript, and supervised the whole project. All authors contributed to the manuscript and approved the submitted version.

## Conflict of Interest

The authors declare that the research was conducted in the absence of any commercial or financial relationships that could be construed as a potential conflict of interest.

## Publisher’s Note

All claims expressed in this article are solely those of the authors and do not necessarily represent those of their affiliated organizations, or those of the publisher, the editors and the reviewers. Any product that may be evaluated in this article, or claim that may be made by its manufacturer, is not guaranteed or endorsed by the publisher.

## References

[B1] Al Ashhab A. Herzberg M. Gillor O. (2014). Biofouling of reverse-osmosis membranes during tertiary wastewater desalination: microbial community composition. *Water Res.* 50 341–349. 10.1016/j.watres.2013.10.044 24231030

[B2] Amann R. I. Ludwig W. Schleifer K. H. (1995). Phylogenetic identification and in situ detection of individual microbial cells without cultivation. *Microbiol. Rev.* 59 143–169. 10.1128/mmbr.59.1.143-169.1995 7535888 PMC239358

[B3] Amato K. R. Yeoman C. J. Kent A. Righini N. Carbonero F. Estrada A. (2013). Habitat degradation impacts black howler monkey (Alouatta pigra) gastrointestinal microbiomes. *ISME J.* 7 1344–1353. 10.1038/ismej.2013.16 23486247 PMC3695285

[B4] Ashie I. N. Smith J. P. Simpson B. K. (1996). Spoilage and shelf-life extension of fresh fish and shellfish. *Crit. Rev. Food Sci. Nutr.* 36 87–121. 10.1080/10408399609527720 8747101

[B5] Boziaris I. S. Parlapani F. F. (2017). “Specific Spoilage Organisms (SSOs) in Fish,” in *The Microbiological Quality of Food*, eds Bevilacqua A. Corbo M. R. Sinigaglia M. (Sawston: Woodhead Publishing), 61–98. 10.3390/foods11030338

[B6] Cai W. Wang Y. Hou Q. Zhang Z. Tang F. Shan C. (2021a). Rice varieties affect bacterial diversity, flavor, and metabolites of zha-chili. *Food Res. Int.* 147:110556. 10.1016/j.foodres.2021.110556 34399533

[B7] Cai W. Wang Y. Ni H. Liu Z. Liu J. Zhong J. A. (2021b). Diversity of microbiota, microbial functions, and flavor in different types of low-temperature Daqu. *Food Res. Int.* 150:110734. 10.1016/j.foodres.2021.110734 34865753

[B8] Cai W. Xue Y. A. Tang F. Wang Y. Yang S. Liu W. (2022). The Depth-Depended Fungal Diversity and Non-depth-Depended Aroma Profiles of Pit Mud for Strong-Flavor Baijiu. *Front. Microbiol.* 12:789845. 10.3389/fmicb.2021.789845 35069486 PMC8770870

[B9] Cao R. Zhao L. Sun H. Liu Q. (2018). Characterization of microbial community in high-pressure treated oysters by high-throughput sequencing technology. *Innovat. Food Sci. Emerg. Technol.* 45 241–248. 10.1016/j.ifset.2017.11.001

[B10] Caporaso J. G. Kuczynski J. Stombaugh J. Bittinger K. Bushman F. D. Costello E. K. (2010). QIIME allows analysis of high-throughput community sequencing data. *Nat. Methods* 7 335–336. 10.1038/nmeth.f.303 20383131 PMC3156573

[B11] Caporaso J. G. Lauber C. L. Walters W. A. Berg-Lyons D. Huntley J. Fierer N. (2012). Ultra-high-throughput microbial community analysis on the Illumina HiSeq and MiSeq platforms. *ISME J.* 6 1621–1624. 10.1038/ismej.2012.8 22402401 PMC3400413

[B12] Chen D. Ci M. Dai R. Chen R. Li T. (2021). Changes in the Microbial Communities of Tiger Frog (Rana tigrina) Meat during Refrigerated Storage. *J. Food Prot.* 84 1136–1140. 10.4315/jfp-20-381 33465236

[B13] Chen H. Liu Z. Shi Y. Ding H. H. (2016). Microbiological analysis and microbiota in oyster: a review. *ISJ* 13 374–388. 10.3389/fimmu.2021.630343 33679773 PMC7930376

[B14] Chen H. Wang M. Yang C. Wan X. Ding H. H. Shi Y. (2019). Bacterial spoilage profiles in the gills of Pacific oysters (Crassostrea gigas) and Eastern oysters (C. virginica) during refrigerated storage. *Food Microbiol.* 82 209–217. 10.1016/j.fm.2019.02.008 31027776

[B15] Chen S. F. Zhou Y. Q. Chen Y. R. Gu J. (2018). fastp: an ultra-fast all-in-one FASTQ preprocessor. *Bioinformatics* 34 884–890. 10.1093/bioinformatics/bty560 30423086 PMC6129281

[B16] Cocolin L. Alessandria V. Dolci P. Gorra R. Rantsiou K. (2013). Culture independent methods to assess the diversity and dynamics of microbiota during food fermentation. *Int. J. Food Microbiol.* 167 29–43. 10.1016/j.ijfoodmicro.2013.05.008 23791362

[B17] Edgar R. C. (2013). UPARSE: highly accurate OTU sequences from microbial amplicon reads. *Nat. Methods* 10:996. 10.1038/nmeth.2604 23955772

[B18] Egerton S. Culloty S. Whooley J. Stanton C. Ross R. P. (2018). The Gut Microbiota of Marine Fish. *Front. Microbiol.* 9:873. 10.3389/fmicb.2018.00873 29780377 PMC5946678

[B19] Han S. Wang Y. Li Y. Shi K. (2021). Investigation of bacterial diversity in Cajanus cajan-planted gangue soil *via* high-throughput sequencing. *Bioengineered* 12 6981–6995. 10.1080/21655979.2021.1976043 34545768 PMC8806674

[B20] Jami M. Ghanbari M. Kneifel W. Domig K. J. (2015). Phylogenetic diversity and biological activity of culturable Actinobacteria isolated from freshwater fish gut microbiota. *Microbiol. Res.* 175 6–15. 10.1016/j.micres.2015.01.009 25662514

[B21] Klindworth A. Pruesse E. Schweer T. Peplies J. Quast C. Horn M. (2013). Evaluation of general 16S ribosomal RNA gene PCR primers for classical and next-generation sequencing-based diversity studies. *Nucleic Acids Res.* 41:e1. 10.1093/nar/gks808 22933715 PMC3592464

[B22] Kuebutornye F. K. A. Abarike E. D. Lu Y. (2019). A review on the application of Bacillus as probiotics in aquaculture. *Fish Shellfish Immunol.* 87 820–828. 10.1016/j.fsi.2019.02.010 30779995

[B23] La Valley K. J. Jones S. Gomez-Chiarri M. Dealteris J. Rice M. (2009). Bacterial community profiling of the eastern oyster (crassostrea virginica): comparison of culture-dependent and culture-independent outcomes. *J. Shellfish Res.* 28 827–835. 10.2983/035.028.0412

[B24] Li H. F. Li Z. J. Qu J. H. Wang J. S. (2017). Bacterial diversity in traditional Jiaozi and sourdough revealed by high-throughput sequencing of 16S rRNA amplicons. *LWT Food Sci. Technol.* 81 319–325. 10.1016/j.lwt.2017.04.007

[B25] Li J. Chen X. Li S. Zuo Z. Zhan R. He R. (2020). Variations of rhizospheric soil microbial communities in response to continuousAndrographis paniculatacropping practices. *Bot. Stud.* 61:18. 10.1186/s40529-020-00295-1 32542518 PMC7295922

[B26] Li M. Chen Z. Qian J. Wei F. Zhang G. Wang Y. (2020). Composition and function of rhizosphere microbiome ofPanax notoginsengwith discrepant yields. *CHIN. Med.* 15:85. 10.1186/s13020-020-00364-4 32793300 PMC7418314

[B27] Li Y.-F. Yang N. Liang X. Yoshida A. Osatomi K. Power D. (2018). Elevated Seawater Temperatures Decrease Microbial Diversity in the Gut of Mytilus coruscus. *Front. Physiol.* 9:839. 10.3389/fphys.2018.00839 30042689 PMC6049046

[B28] Liu X. Teixeira J. S. Ner S. Ma K. V. Petronella N. Banerjee S. (2020). Exploring the Potential of the Microbiome as a Marker of the Geographic Origin of Fresh Seafood. *Front. Microbiol.* 11:696. 10.3389/fmicb.2020.00696 32362885 PMC7181054

[B29] Madigan T. L. Bott N. J. Torok V. A. Percy N. J. Carragher J. F. Lopes M. A. D. (2014). A microbial spoilage profile of half shell Pacific oysters (Crassostrea gigas) and Sydney rock oysters (Saccostrea glomerata). *Food Microbiol.* 38 219–227. 10.1016/j.fm.2013.09.005 24290646

[B30] Magoc T. Salzberg S. L. (2011). FLASH: fast length adjustment of short reads to improve genome assemblies. *Bioinformatics* 27 2957–2963. 10.1093/bioinformatics/btr507 21903629 PMC3198573

[B31] Milan M. Carraro L. Fariselli P. Martino M. E. Cavalieri D. Vitali F. (2018). Microbiota and environmental stress: how pollution affects microbial communities in Manila clams. *Aqua. Toxicol.* 194 195–207. 10.1016/j.aquatox.2017.11.019 29202271

[B32] Naum M. Brown E. W. Mason-Gamer R. J. (2008). Is 16S rDNA a reliable phylogenetic marker to characterize relationships below the family level in the *Enterobacteriaceae*? *J. Mol. Evol.* 66 630–642. 10.1007/s00239-008-9115-3 18504519

[B33] Nie H. Jiang L. Huo Z. Liu L. Yang F. Yan X. (2016). Transcriptomic responses to low temperature stress in the Manila clam. *Ruditapes philippinarum*. *Fish Shellfish Immunol.* 55 358–366. 10.1016/j.fsi.2016.06.008 27288255

[B34] Niu K.-M. Lee B.-J. Kothari D. Lee W.-D. Hur S.-W. Lim S.-G. (2020). Dietary effect of low fish meal aquafeed on gut microbiota in olive flounder (Paralichthys olivaceus) at different growth stages. *Microbiologyopen* 9:e992. 10.1002/mbo3.992 31925997 PMC7066472

[B35] Odeyemi O. A. Burke C. M. Bolch C. C. J. Stanley R. (2019). Spoilage microbial community profiling by 16S rRNA amplicon sequencing of modified atmosphere packaged live mussels stored at 4 degrees C. *Food Res. Int.* 121 568–576. 10.1016/j.foodres.2018.12.017 31108782

[B36] Pan Z. Li L. Shen Z. Chen Y. Li M. (2018). Characterization of the Microbiota in Air- or Vacuum-Packed Crisp Grass Carp (Ctenopharyngodon idella C. et V.) Fillets by 16S rRNA PCR-Denaturing Gradient Gel Electrophoresis and High-Throughput Sequencing. *J. Food Prot.* 81 1022–1029. 10.4315/0362-028x.Jfp-17-498 29761724

[B37] Pereira G. D. V. da Cunha D. G. Pedreira Mourino J. L. Rodiles A. Jaramillo-Torres A. Merrifield D. L. (2017). Characterization of microbiota in Arapaima gigas intestine and isolation of potential probiotic bacteria. *J. Appl. Microbiol.* 123 1298–1311. 10.1111/jam.13572 28833934

[B38] Prathiviraj R. Rajeev R. Fernandes H. Rathna K. Lipton A. N. Selvin J. (2021). A gelatinized lipopeptide diet effectively modulates immune response, disease resistance and gut microbiome in Penaeus vannamei challenged with Vibrio parahaemolyticus. *Fish Shellfish Immunol.* 112 92–107. 10.1016/j.fsi.2021.02.018 33675990

[B39] Ramirez C. Soledad Gutierrez M. Venegas L. Sapag C. Araya C. Caruffo M. (2022). Microbiota composition and susceptibility to florfenicol and oxytetracycline of bacterial isolates from mussels (Mytilus spp.) reared on different years and distance from salmon farms. *Environ. Res.* 204:112068. 10.1016/j.envres.2021.112068 34547250

[B40] Schmitt P. Rosa R. D. Duperthuy M. de Lorgeril J. Bachere E. Destoumieux-Garzon D. (2012). The antimicrobial defense of the Pacific oyster, Crassostrea gigas. How diversity may compensate for scarcity in the regulation of resident/pathogenic microflora. *Front. Microbiol.* 3:160. 10.3389/fmicb.2012.00160 22783227 PMC3390580

[B41] Segata N. Izard J. Waldron L. Gevers D. Miropolsky L. Garrett W. S. (2011). Metagenomic biomarker discovery and explanation. *Genome Biol.* 12:R60. 10.1186/gb-2011-12-6-r60 21702898 PMC3218848

[B42] Shi F. Zi Y. Lu Z. Li F. Yang M. Zhan F. (2020). Bacillus subtilis H2 modulates immune response, fat metabolism and bacterial flora in the gut of grass carp (Ctenopharyngodon idellus). *Fish Shellfish Immunol.* 106 8–20. 10.1016/j.fsi.2020.06.061 32717323

[B43] Stackebrandt E. Goebel B. M. (1994). Taxonomic note: a place for DNA-DNA reassociation and 16S rRNA sequence analysis in the present species definition in bacteriology. *Int. J. Syst. Bacteriol.* 44, 846–849.

[B44] Tan L. T.-H. Chan K.-G. Lee L.-H. Goh B.-H. (2016). Streptomyces Bacteria as Potential Probiotics in Aquaculture. *Front. Microbiol.* 7:79. 10.3389/fmicb.2016.00079 26903962 PMC4742533

[B45] Tan Y. Fang L. Qiu M. Huo Z. Yan X. (2020). Population genetics of the Manila clam (Ruditapes philippinarum) in East Asia. *Sci. Rep.* 10:21890. 10.1038/s41598-020-78923-w 33318552 PMC7736867

[B46] Trabal N. Mazon-Suastegui J. M. Vazquez-Juarez R. Asencio-Valle F. Morales-Bojorquez E. Romero J. (2012). Molecular Analysis of Bacterial Microbiota Associated with Oysters (Crassostrea gigas and Crassostrea corteziensis) in Different Growth Phases at Two Cultivation Sites. *Microb. Ecol.* 64 555–569. 10.1007/s00248-012-0039-5 22450510

[B47] Wang Q. Garrity G. M. Tiedje J. M. Cole J. R. (2007). Naive Bayesian classifier for rapid assignment of rRNA sequences into the new bacterial taxonomy. *Appl. Environ. Microbiol.* 73 5261–5267. 10.1128/aem.00062-07 17586664 PMC1950982

[B48] Wang S.-T. Meng X.-Z. Dai Y.-F. Zhang J.-H. Shen Y. Xu X.-Y. (2021). Characterization of the intestinal digesta and mucosal microbiome of the grass carp (Ctenopharyngodon idella). *Comp. Biochem. Physiol. Part D Genomics Proteomics* 37:100789. 10.1016/j.cbd.2021.100789 33465759

[B49] Yang C. Che Y. Qi Y. Liang P. Song C. (2017). High-Throughput Sequencing of Viable Microbial Communities in Raw Pork Subjected to a Fast Cooling Process. *J. Food Sci.* 82 145–153. 10.1111/1750-3841.13566 27871121

[B50] Zhang C. Zheng X. Ren X. Li Y. Wang Y. (2019). Bacterial diversity in gut of large yellow croaker Larimichthys crocea and black sea bream Sparus macrocephalus reared in an inshore net pen. *Fish. Sci.* 85 1027–1036. 10.1007/s12562-019-01349-5

[B51] Zhao H. Zhang S. (2016). Identification of Jiaozhou Bay Clams (Ruditapes philippinarum) by Multi-element Fingerprinting Technique. *Food Anal. Methods* 9 2691–2699. 10.1007/s12161-016-0461-2

[B52] Zhukova N. V. Eliseikina M. G. Balakirev E. S. Ayala F. J. (2022). Multiple bacterial partners in symbiosis with the nudibranch mollusk Rostanga alisae. *Sci. Rep.* 12:169. 10.1038/s41598-021-03973-7 34997021 PMC8742107

