# Supplementary material for: Exploring the Dynamic of Bacterial Communities in Manila Clam (Ruditapes philippinarum) During Refrigerated Storage
Source: Front Microbiol. 2022 May 18;13:882629. doi: 10.3389/fmicb.2022.882629 (PMC9158497; doi:10.3389/fmicb.2022.882629)
Supplement: Supplementary file 1 [file Table_1.DOCX]

Table S1. Alpha diversity analysis at OTU level.

| Diversity estimator | Day 0 | Day 1 | Day 3 |
| --- | --- | --- | --- |
| Sobs | 2058.70±38.50 | 1680.00±1007.00 | 1694.70±491.60 |
| Ace | 2473.90±67.57 | 2044.60±1183.30 | 2018.00±681.69 |
| Chao | 2467.20±87.16 | 2054.20±1172.80 | 2034.40±696.37 |
| Shannon | 6.32±0.02 | 5.66±1.23 | 5.97±0.29 |
| Simpson | 0.005±0.00 | 0.016±0.016 | 0.009±0.00 |
